# Clinically Silent Local Recurrence of Breast Cancer Detected in a Mastectomy Scar During DIEP Flap Reconstruction: A Case Report

**DOI:** 10.1002/ccr3.72701

**Published:** 2026-06-10

**Authors:** David Tesina, Tomas Herma, Jiri Bayer, Radoslav Matej, Andrej Sukop, Pavla Ticha

**Affiliations:** ^1^ Department of Plastic Surgery, Kralovske Vinohrady University Hospital and Third Faculty of Medicine Charles University Praha Czech Republic; ^2^ Department of Anatomy, Second Faculty of Medicine Charles University Praha Czech Republic; ^3^ Department of Pathology, Kralovske Vinohrady University Hospital and Third Faculty of Medicine Charles University Praha Czech Republic

**Keywords:** breast cancer, breast reconstruction, DIEP flap, histopathological examination, local cancer recurrence, mastectomy scar

## Abstract

This case report presents a rare event of clinically silent local breast cancer recurrence detected during delayed breast reconstruction with a DIEP flap in a patient considered to be in complete remission. The recurrence was not detected by preoperative imaging or serum tumor markers but was unexpectedly identified in the excised mastectomy scar during histopathological examination. This case underscores the diagnostic importance of routine histopathological examination of mastectomy scars in patients assessed to be in complete remission and undergoing secondary procedures such as autologous breast reconstruction. This is the first reported case of breast cancer recurrence detected in a mastectomy scar excised during DIEP flap reconstruction, which subsequently required flap elevation and oncologic re‐resection. Furthermore, unlike in other published cases, the technically challenging re‐resection was performed only 23 days after the initial reconstructive surgery.

## Key Clinical Message

Histopathological examination of the mastectomy scar during autologous breast reconstruction can detect clinically silent breast cancer recurrence undetectable by imaging or serum markers. This rare but critical finding underscores the importance of mastectomy scar excision, and consequently reconstruction technique choice, even in patients considered in complete remission, to enable timely intervention.

## Graphical abstract

Histopathological analysis of the mastectomy scar during delayed autologous breast reconstruction revealed clinically occult local breast cancer recurrence. The patient subsequently underwent tumor re‐resection with preservation of the microvascular pedicle and repositioning of the DIEP flap.

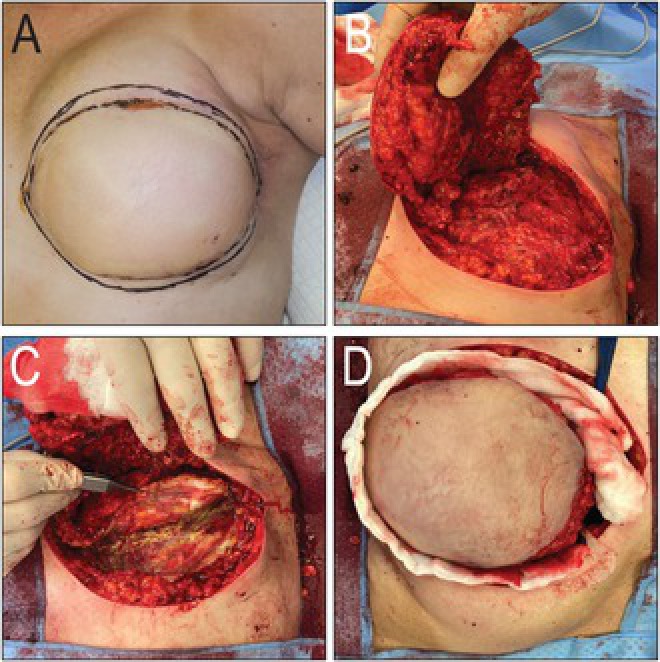

## 1 Introduction

Local recurrence of breast cancer following mastectomy and either immediate or delayed autologous reconstruction is a rare yet clinically significant event. Most recurrences occur in the superficial tissues of the reconstructed breast and are detected through physical examination or imaging [1]. As our case illustrates, despite regular oncologic follow‐up, which includes imaging and tumor marker monitoring, not all recurrences are clinically or radiologically evident. In rare instances, recurrence may go undetected by conventional methods such as PET/CT, ultrasound, or serum tumor markers. We present the case of a patient in clinical and radiologic remission who was diagnosed with asymptomatic local recurrence only after histopathological evaluation of a routinely excised mastectomy scar during breast reconstruction, with a need for early re‐resection of the tumor in the terrain of fresh free‐flap surgery. This case highlights the potential role of scar excision and histopathological analysis in the early detection of recurrence in patients with a history of breast cancer with histologic subtypes that exhibit limited FDG PET/CT detectability.

## 2 Case History and Examination

A 51‐year‐old woman was diagnosed with invasive breast carcinoma of no special type (IBC‐NST) in the left breast, staged as T3‐4, N1, M0, with ER 70%, PR 90%, HER2/neu 3+, and Ki‐67 index 55%. The patient underwent a left‐sided mastectomy and axillary dissection, followed by neoadjuvant chemotherapy, radiotherapy, and hormonal therapy with aromatase inhibitors. Genetic testing for cancer‐related mutations returned negative results. The patient has remained in clinical and radiological remission. A PET/CT scan performed three years after the mastectomy showed no evidence of recurrence or new pathology. Later that year, the oncological check‐up revealed normal levels of serum tumor markers, along with a normal breast ultrasound and mammography. The patient was referred by her oncologist for reconstructive surgery.

## 3 Methods

**FIGURE 1:**
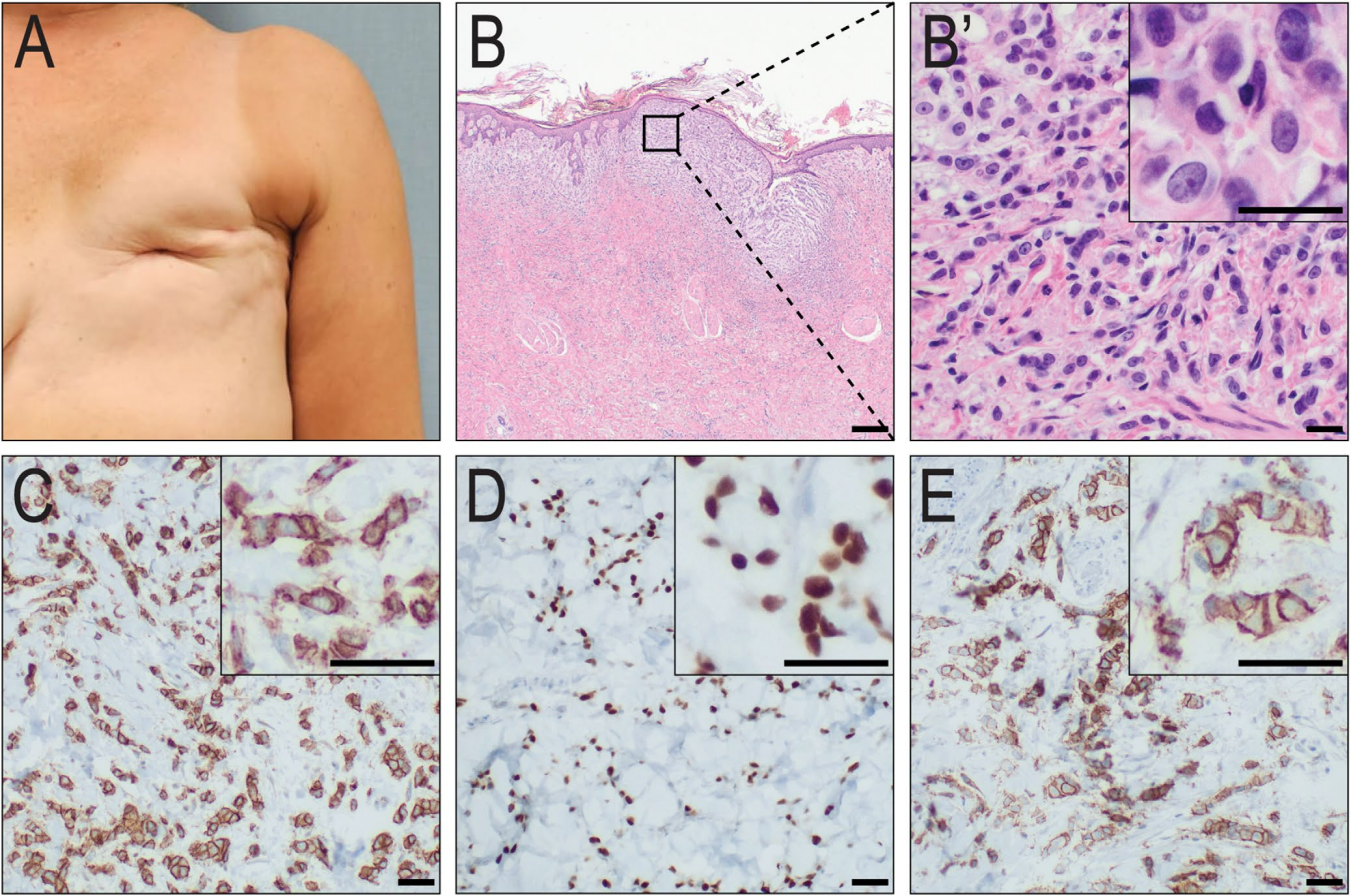
Histopathological analysis of a mastectomy scar. (A) Preoperative photographs of the mastectomy scar before delayed DIEP flap reconstruction, showing no visible signs of recurrence. (B, B′) Representative images of formalin‐fixed paraffin embedded tissue sections from mastectomy scar specimen excised during DIEP flap reconstruction, stained with hematoxylin and eosin. The sections revealed invasive carcinoma cells with a lobular growth pattern and moderate nuclear atypia. Immunostaining analysis of the tumor cells showed positive staining for (C) E‐cadherin and strong expression of (D) estrogen receptor and (E) HER2/neu. (Scale bars: B: 200 μm, B′: 20 μm, C–E: 50 μm).

A DIEP flap reconstruction was performed in the same month as the most recent oncology follow‐up. The DIEP flap was selected as it utilizes autologous tissue to provide a natural aesthetic result and avoids implant‐related complications. Moreover, the radical excision of the previous mastectomy scar is advantageous for improving local soft‐tissue conditions, especially where the scar is tethered to the underlying pectoralis muscle and ribs. During the procedure, the mastectomy scar (Figure 1A) was excised for histopathological examination, as is routine at our institution. Unexpectedly, histopathological examination using hematoxylin and eosin staining revealed a lobular‐pattern, moderately differentiated IBC‐NST within the scar tissue, measuring 30 mm in the largest dimension and characterized by moderate nuclear atypia (Figure 1B,B'). Focal ulceration of the superficial dermis was observed. The tumor extended to the base of the excision and reached the margin centrally. Immunohistochemistry showed retained E‐cadherin expression (Figure 1C). The tumor was staged pT4 with ER 95% (Figure 1D), PR 0%, HER2/neu 3+ (Figure 1E), with a Ki‐67 index of up to 20%, and a G2 differentiation grade.

**FIGURE 2:**
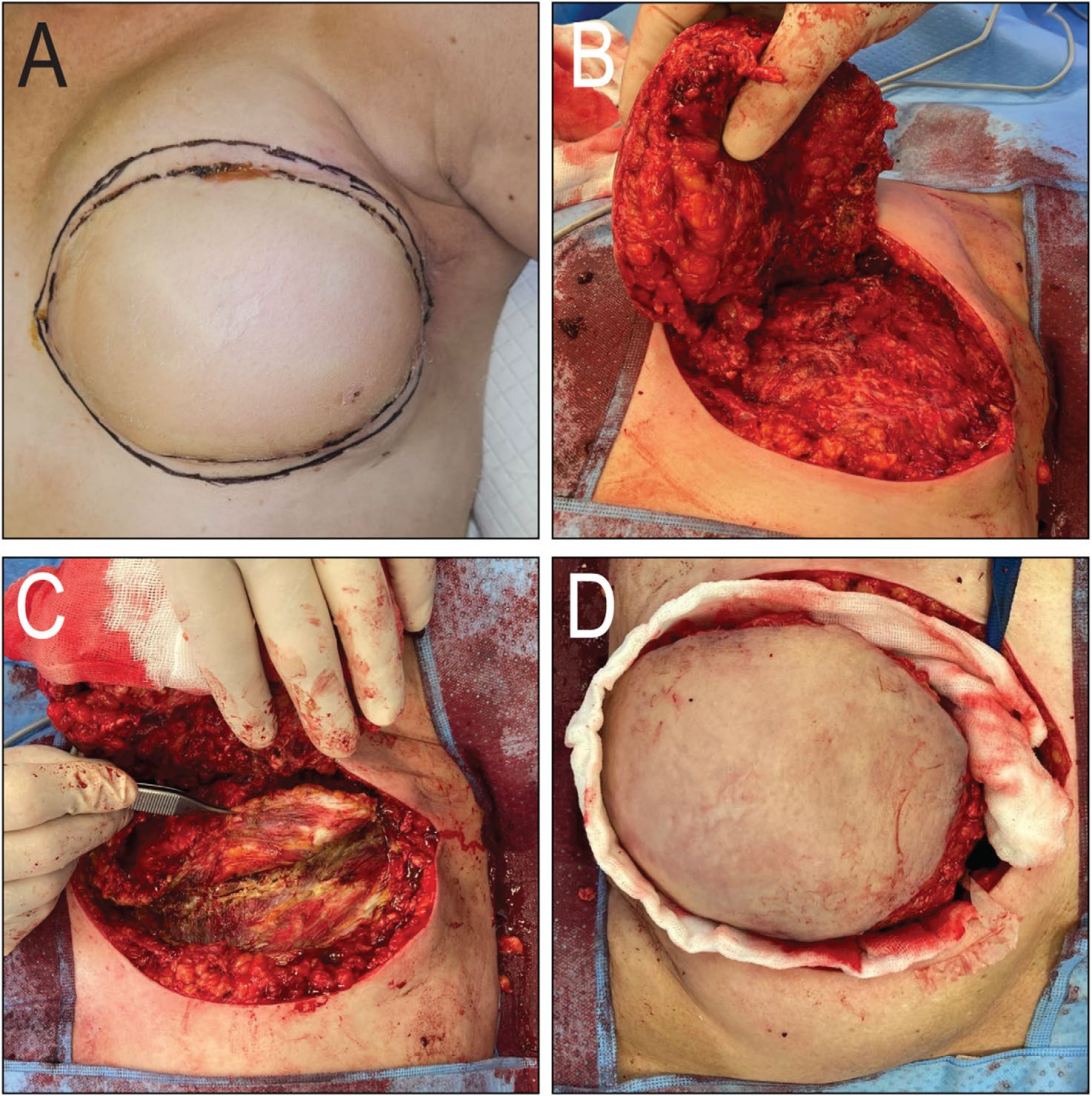
Tumor re‐resection and flap reposition. (A) Preoperative clinical planning marking the required margins for the re‐operation 23 days after the initial reconstruction. (B) Intraoperative photograph showing the elevation of the DIEP flap to access the deeper tissue planes. (C) View of the radical re‐resection of the tissue bed with careful preservation of the microvascular pedicle supplying the flap. (D) Immediate postoperative result following flap repositioning into the new tissue bed.

The case was presented at multidisciplinary seminar, and the patient was indicated for reoperation on post‐reconstruction day 23 (Figure 2A). The flap was mobilized without disrupting its microvascular pedicle (Figure 2B). Two tissue specimens were excised: one from the circumferential skin margins around the previous flap inset of size 18 x 6 cm and the other from the deep resection plane that included the underlying pectoralis major muscle of size 10 x 9.5 cm (Figure 2C). The flap was repositioned and utilized to re‐cover the defect (Figure 2D). Postoperatively, the patient received intravenous ampicillin/sulbactam, which was later switched to oral administration and discontinued on post‐reconstruction day 29. A minor wound infection occurred in the flap area, which was managed conservatively.

## 4 Results

**FIGURE 3:**
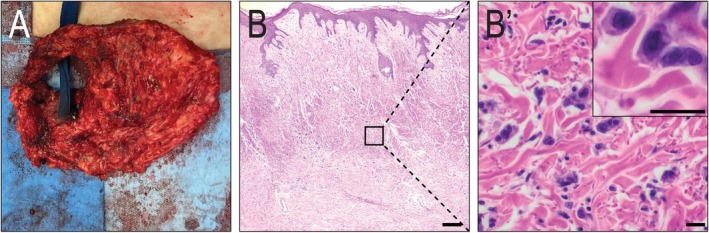
Re‐resection specimen examination. (A) Macroscopic view of the deep resection plane specimen, including the pectoralis major muscle segment. (B) Representative images of formalin‐fixed paraffin embedded tissue sections from a re‐resection specimen stained with hematoxylin and eosin, showing residual tumor cells (B, B′) (Scale bars: B: 200 μm, B′: 20 μm).

Histopathological examination of the specimen, including the circumferential skin margins around the previous flap inset, revealed residual tumor measuring 4 × 2 mm. Examination of the deep resection plane specimen, which included the underlying pectoralis major muscle (Figure 3A), showed residual tumor measuring 1 × 1 mm (Figure 3B,B'). Both resections were complete (R0). The patient was subsequently referred to her oncologist for continued follow‐up care.

## 5 Discussion

Local recurrence of breast cancer following mastectomy and autologous reconstruction is an uncommon yet significant event. Studies have reported approximately 2% to 7% local recurrence rates in patients undergoing these procedures [2]. Such recurrences often present within the first few years post‐surgery, emphasizing the importance of vigilant long‐term follow‐up. The detection of clinically silent recurrences presents a diagnostic challenge.

There has been an almost 40‐year‐long debate about whether the routine histological examination of mastectomy scars should be part of breast reconstruction surgeries [3]. The advocates of this practice point out the oncological benefits of potential early intervention [4], the relatively low cost of the histological examination [5], and the difficulty in distinguishing between benign and potentially malignant scar tissue [6]. However, more than twice as many studies oppose the practice due to concerns about time and cost‐effectiveness; only 0.19% of post‐mastectomy scars have a local recurrence present [7], and the proportion of local skin recurrences in the scar is relatively low compared to the rest of the breast skin [8]. The preventive impact is questionable, as 2.21% of the post‐reconstruction patients developed a recurrence not related to the scar during the follow‐up period. Those authors more often suggest a more selective use of histopathological examination based on potential risk factors, including tumor size, margins, TNM score, grade, receptor status, and patient age, among others [7]. Admittedly, from a cost–benefit perspective, routine histopathological analysis of every scar may be debated, given the low recurrence rate and the associated laboratory costs. However, in high‐risk patients or those with aggressive primary tumor biology, the clinical benefit of early detection can significantly outweigh these costs, as identifying an otherwise occult malignancy is critical for patient survival and immediate management.

In our case, the incidental finding of a 30 mm invasive carcinoma within the scar tissue highlights the potential value of this practice. The possible mechanisms behind the development of local recurrence or new cancer after mastectomy are not well understood. Explanations may include residual cancer, tumor spillage during the initial operation, or hematogenous tumor spread. Moreover, it should be noted that all forms of mastectomy, whether radical, modified radical, or skin‐sparing, might leave some residual breast tissue [9], which can also potentially progress to malignancy over time. The clinical presentation of locally recurrent breast carcinoma varies and may include palpable nodules along the suture line, skin thickening, or an erythematous rash [2]. Although rare, some recurrences have been reported deep to the pectoralis major muscle and are considered chest wall recurrences [10]. Most recurrences, however, occur within the superficial soft tissue of the reconstructed breast and are typically identified through clinical examination [1, 10].

Importantly, no imaging studies or serum tumor markers detected this tumor prior to the reconstruction. While 18F‐FDG PET/CT and serum tumor markers such as CA 15–3 are often employed in follow‐up, their sensitivity remains limited. A substantial proportion of patients with confirmed recurrence may present with normal CA 15–3 levels [11]. Moreover, the detectability of breast cancer lesions on FDG PET/CT depends on the histologic subtype and receptor status of the cancer. The FDG uptake is typically lower in tumors with hormone receptor positivity, lower grade, lobular pattern [12], and retained expression of epithelial adhesive markers, such as E‐cadherin [13], all of which were characteristics observed in this case. That, in turn, may reduce PET/CT sensitivity in these cases, underscoring the limitations of current surveillance strategies and supporting a more cautious and individualized approach. While advancing imaging technologies remain a major focus for oncological surveillance, this case confirms that histopathological examination still has an important role in detecting occult recurrences, particularly in small‐volume disease or in subtypes with low metabolic activity. Although histopathological analysis remains indispensable in our case, this should not detract from the continued development and clinical validation of high‐resolution imaging or liquid biopsy techniques, which may eventually offer superior sensitivity for identifying occult recurrences non‐invasively. Furthermore, the choice of a DIEP flap reconstruction proved crucial; although the scar appeared clinically and macroscopically unremarkable during surgery, the radical excision required to improve the local soft‐tissue bed for the flap enabled the subsequent histopathological analysis that unexpectedly revealed the malignancy. This underscores that without the specific surgical requirements of autologous reconstruction, such a clinically silent recurrence would likely have remained undetected.

As demonstrated, the choice of reconstruction technique played a pivotal role in detecting the recurrence in our patient. Utilizing a DIEP flap required excising the previous mastectomy scar, which led to the identification of the tumor. To put this into context, of the 11 published studies, 7 cases of recurrence of breast cancer in post‐mastectomy scars out of 3754 scar examinations were published [7]. Those cases included reconstruction with a latissimus dorsi flap and tissue expander [5], a latissimus dorsi flap and breast implant, tissue expander [4, 14], and delayed excision of the scar 6 months after reconstruction with a TRAM flap [6]. In our case, the positive histopathological finding in the scar led to a technically challenging re‐resection of the skin margins and elevation of the flap with complete re‐resection of the deep resection plane, including the pectoralis major muscle, with preservation of the vascular pedicle, only 23 days after the reconstruction. To our knowledge, no similar case has been reported before.

Conversely, implant‐based reconstructions, typically approached through the inframammary fold, might not involve scar excision, potentially leaving residual disease undetected. Therefore, when planning the type of breast reconstruction, it is important to consider that if the surgical approach does not involve excision of the mastectomy scar, such as in subpectoral implant‐based reconstructions, an asymptomatic recurrence may remain undiagnosed.

Another possibility that must be considered is that the initial tumorectomy was incomplete and that a limitation occurred during histopathological evaluation. Specifically, the margins may have been interpreted as tumor‐free due to sampling constraints or diagnostic limitations, when in fact, the resection may not have encompassed the full extent of the tumor.

This case demonstrates that routine histopathological analysis of excised mastectomy scars during autologous breast reconstruction can uncover clinically silent local recurrence. Although rare, such findings can have a significant impact on patient prognosis and management. Furthermore, in this case, the choice of reconstructive technique involving scar excision was potentially life‐saving, underscoring the importance of careful surgical planning even in patients considered to be in complete remission.

## Author Contributions

**David Tesina:** conceptualization, data curation, methodology, resources, writing – original draft, writing – review and editing. **Tomas Herma:** formal analysis, investigation, writing – original draft, writing – review and editing. **Jiri Bayer:** conceptualization, data curation, investigation, resources, writing – original draft, writing – review and editing. **Radoslav Matej:** visualization, writing – original draft, writing – review and editing. **Andrej Sukop:** supervision, writing – original draft, writing – review and editing. **Pavla Ticha:** formal analysis, funding acquisition, methodology, visualization, writing – original draft, writing – review and editing.

## Funding

This work was supported by Univerzita Karlova v Praze (Grant PRIMUS/25/MED/002).

## Ethics Statement

All procedures performed in this study involving human participants were in accordance with the ethical standards of the institutional and/or national research committee and with the Helsinki Declaration and its later amendments, or comparable ethical standards. This case report has been prepared in accordance with the CARE guidelines.

## Consent

Written informed consent was obtained from the patient to publish this report in accordance with the journal's patient consent policy.

## Conflicts of Interest

The authors declare no conflicts of interest.

## Data Availability

Data sharing not applicable to this article as no datasets were generated or analysed during the current study.

## References

[1] J. H. Joo , Y. Ki , W. Kim , et al., “Pattern of Local Recurrence After Mastectomy and Reconstruction in Breast Cancer Patients: A Systematic Review,” Gland Surgery 10, no. 6 (2021): 2037–2046, 10.21037/gs-21-15. 34268088 PMC8258883

[2] W. Shinzaki , H. Manabe , M. Kubota , et al., “Breast Cancer Local Recurrence After Mastectomy With Immediate Latissimus Dorsi Myocutaneous Flap Reconstruction: A Case Report,” SAGE Open Medical Case Reports 6, no. 11 (2023): 2050313X231177510, 10.1177/2050313X231177510. PMC10265333 37325163

[3] M. S. Granick , R. W. Bragdon , and D. C. Hanna , “Recurrent Breast Carcinoma at the Time of Breast Reconstruction,” Annals of Plastic Surgery 18, no. 1 (1987): 69–70, 10.1097/00000637-198701000-00014. 3827134

[4] R. M. Warner , D. L. Wallace , N. A. Ferran , et al., “Mastectomy Scars Following Breast Reconstruction: Should Routine Histologic Analysis Be Performed?,” Plastic and Reconstructive Surgery 123, no. 4 (2009): 1141–1147, 10.1097/PRS.0b013e31819f25d5. 19337082

[5] G. J. Zambacos , P. A. Wilson , D. Miminas , and R. J. Morris , “Routine Histological Examination of the Mastectomy Scar at the Time of Breast Reconstruction,” British Journal of Plastic Surgery 58, no. 1 (2005): 122, 10.1016/j.bjps.2004.06.030. 15629184

[6] M. Y. Nahabedian , “Routine Histologic Examination of 728 Mastectomy Scars: Did It Benefit Our Patients?,” Plastic and Reconstructive Surgery 120, no. 1 (2007): 353, 10.1097/01.prs.0000264570.56306.50. 17572595

[7] O. Berger and R. Talisman , “Histologic Examination of Mastectomy Scars During Breast Reconstruction: A Systematic Review,” Plastic and Reconstructive Surgery Global Open 12, no. 5 (2024): e5847, 10.1097/GOX.0000000000005847. 38798931 PMC11124693

[8] O. Kaidar‐Person , P. Poortmans , B. V. Offersen , et al., “Spatial Location of Local Recurrences After Mastectomy: A Systematic Review,” Breast Cancer Research and Treatment 183, no. 2 (2020): 263–273, 10.1007/s10549-020-05774-4. 32661665

[9] M. J. Napierała , W. Olszewski , M. Rosińska , et al., “Residual Breast Tissue After Four Types of Mastectomy: A Prospective, Comparative Study,” Annals of Surgical Oncology 32, no. 8 (2025): 5467–5476, 10.1245/s10434-025-17350-5. 40272669

[10] H. N. Langstein , M. H. Cheng , S. E. Singletary , et al., “Breast Cancer Recurrence After Immediate Reconstruction: Patterns and Significance,” Plastic and Reconstructive Surgery 111, no. 2 (2003): 712–720, 10.1097/01.prs.0000041441.42563.95. 12560692

[11] M. J. Duffy , D. Evoy , and E. W. McDermott , “CA 15‐3: Uses and Limitation as a Biomarker for Breast Cancer,” Clinica Chimica Acta 411, no. 23–24 (2010): 1869–1874, 10.1016/j.cca.2010.08.039. 20816948

[12] G. A. Ulaner , “PET/CT for Patients With Breast Cancer: Where Is the Clinical Impact?,” American Journal of Roentgenology 213, no. 2 (2019): 254–265, 10.2214/ajr.19.21177. 31063423

[13] K. Higashi , Y. Ueda , M. Shimasaki , et al., “High FDG Uptake on PET Is Associated With Negative Cell‐To‐Cell Adhesion Molecule E‐Cadherin Expression in Lung Adenocarcinoma,” Annals of Nuclear Medicine 31, no. 8 (2017): 590–595, 10.1007/s12149-017-1187-y. 28677069

[14] M. Alam , C. Kiely , S. H. Shah , C. Lawlor , and M. O'Donnell , “Mastectomy Scar Histopathology of Limited Clinical Value,” Annals of Plastic Surgery 57, no. 4 (2006): 374–375, 10.1097/01.sap.0000237565.39876.f9. 16998326

